# Dr. Wm. H. Pleuthner

**Published:** 1901-02

**Authors:** 


Dr. Wm. H. Pleuthner, of Buffalo, died suddenly January 17, 1901, aged 32 years. He was an alumnus of the University of Buffalo, class of '91, and had been engaged in the practice of his profession in this city since graduation. Dr. Pleuthner was a native of Buffalo and is survived by a widow and a brother.

